# Comparative Antioxidant Profiling of Phenolic Acids and Flavonoids: Assay-Resolved Structure–Activity Relationships Under Harmonized In Vitro Conditions

**DOI:** 10.3390/molecules31091478

**Published:** 2026-04-29

**Authors:** Zafer Ömer Özdemir, Merve Soy, Sibel Ataseven, Ayşenur Özer, Mahfuz Elmastaş

**Affiliations:** 1 Department of Analytical Chemistry, Faculty of Hamidiye Pharmacy, University of Health Sciences Türkiye, Istanbul 34668, Türkiye; 2 Department of Pharmacognosy, Institute of Hamidiye Health Sciences, University of Health Sciences Türkiye, Istanbul 34668, Türkiye; 3 Department of Biochemistry, Faculty of Hamidiye Pharmacy, University of Health Sciences Türkiye, Istanbul 34668, Türkiye

**Keywords:** phenolic acids, flavonoids, antioxidant profiling, structure–activity relationship, medicinal chemistry, scaffold ranking, ABTS, DPPH, reducing power

## Abstract

Phenolic acids and flavonoids remain attractive redox-active scaffolds in medicinal chemistry, where they are widely used for early-stage prioritization and intrinsic reactivity ranking. However, direct comparisons under harmonized conditions remain scarce, limiting structure-based assessment. Here, a structurally diverse panel of hydroxybenzoic acids, hydroxycinnamic acids, flavonoids, a flavanone, and synthetic comparators was profiled using Folin–Ciocalteu response, ABTS radical cation scavenging, DPPH radical scavenging, and reducing power assays. The data reveal pronounced assay dependence alongside clear structure–activity trends. Gallic acid showed the strongest DPPH scavenging (half-maximal inhibitory concentration, IC_50_ = 4.45 µmol/L) and reducing power (17.26 µmol TE/mg), while quercetin was consistently active across all four endpoints. Eriocitrin (IC_50_ = 2.47 µmol/L) and rutin (IC_50_ = 2.66 µmol/L) were particularly effective in the ABTS assay, showing that glycosylation does not abolish cation-radical scavenging. Lipinski’s Rule of Five and Veber oral-bioavailability criteria place these findings within a drug-likeness context. The results also highlight the limitations of the Folin–Ciocalteu assay as a standalone measure of total phenolic content, since its response depends strongly on hydroxylation density. Rather than asserting therapeutic efficacy, this work provides a harmonized comparative dataset identifying phenolic substructures with the strongest and most consistent redox activity, together with the structural drivers underlying these patterns.

## 1. Introduction

Oxidative stress is generally defined as an imbalance in which reactive oxygen species exceed the capacity of endogenous defense systems to control or repair oxidative damage. Contemporary biomedical research no longer treats it as nonspecific radical damage, but as an imbalance closely linked to chronic inflammation, neurodegeneration, cardiometabolic dysfunction, and other disease-relevant pathways. Small organic molecules that modulate radical chemistry therefore remain of interest as bioactive agents, chemical probes, and starting points for lead optimization [1,2,3,4]. Beyond their redox chemistry, many of these scaffolds interact with inflammation-related targets such as cyclooxygenase, lipoxygenase, and NF-κB signaling components. Antioxidant activity should therefore be viewed not as a standalone therapeutic endpoint but as a chemical property operating alongside anti-inflammatory mechanisms. The two processes are mechanistically intertwined: reactive oxygen species promote IκB degradation and NF-κB nuclear translocation, while inflammatory signaling in turn amplifies ROS generation through NADPH oxidase and mitochondrial dysfunction. Antioxidant profiling thus complements, rather than substitutes for, target-based anti-inflammatory evaluation. Within this framework, profiling purified single compounds remains valuable, as it captures intrinsic redox reactivity without the confounding effects of plant matrices, metabolism, or formulation [5,6,7].

Among natural redox-active molecules, phenolic acids and flavonoids are of particular interest because their antioxidant behavior is governed by well-defined structural features. These compounds can participate in hydrogen atom transfer (HAT), single-electron transfer (SET), radical stabilization through extended conjugation and resonance delocalization, and, in some cases, metal-chelating processes [8,9]. The HAT mechanism involves the direct transfer of a hydrogen atom from the phenolic O–H group to a free radical, whereas the SET pathway proceeds through electron donation to neutralize radical species; both routes depend critically on the bond dissociation enthalpy and ionization potential of the phenolic substrate [10,11]. Radical stabilization is further facilitated by ortho- and para-hydroxyl substitution patterns that allow extensive resonance delocalization of the resulting phenoxyl radical, while metal chelation—particularly of redox-active transition metals such as Fe^2+^/Fe^3+^ and Cu^+^/Cu^2+^—can prevent Fenton-type radical generation [12,13]. These compound families should not, however, be treated as a chemically uniform class. Hydroxybenzoic acids and hydroxycinnamic acids differ in aromatic substitution and conjugation, whereas flavonoids possess a more complex C6–C3–C6 framework in which B-ring hydroxylation, C2=C3 unsaturation, 4-oxo functionality, ring planarity, and glycosylation can shift reactivity substantially. As a result, two compounds that look chemically similar can behave very differently depending on the radical system, solvent, or mechanistic focus of the assay [11,14].

This issue is central to both phytochemical interpretation and medicinal-chemistry translation. While recent extract-based studies frequently correlate phenolic content with antioxidant activity across various solvent systems [15], antioxidant claims are often inferred from one or two assays without direct comparison of purified compounds under identical conditions. Although substantial evidence on the antioxidant activity of individual polyphenolic compounds exists from DPPH (2,2-diphenyl-1-picrylhydrazyl), ABTS [2,2′-azino-bis(3-ethylbenzothiazoline-6-sulfonic acid)], and FRAP (Ferric reducing antioxidant power) studies, these data are typically generated under heterogeneous conditions across different laboratories. This heterogeneity makes inter-study comparisons unreliable and obscures structure–activity relationships. As a result, it remains difficult to distinguish broad-spectrum redox-active scaffolds from compounds whose apparent potency is strongly method-dependent. The novelty of the present work therefore does not reside in demonstrating that polyphenols are antioxidants—a well-established fact—but in providing a single, internally consistent, multi-assay benchmark that enables direct, ranking-level comparison of 21 structurally diverse scaffolds under identical experimental conditions. Such harmonized datasets—in which all compounds are measured in parallel within a single analytical session using a panel that spans electron-transfer, hydrogen-atom-transfer, and cation-radical scavenging chemistries—are rare in the literature yet essential for reliable scaffold prioritization.

Recent work has reinforced the need for direct compound-level comparison. Unified DPPH-based studies have shown that antioxidant capacity among phenols varies sharply with substitution pattern and ionization-related properties, while theoretical models of flavonoid oxidation further indicate that electronic structure and oxidation potential are tightly linked to observed activity [11,14]. Likewise, simulated digestion studies have shown that phenolic subclasses do not respond uniformly under the same physicochemical conditions, underscoring the need for direct comparative profiling rather than broad generalization [4].

A second complication is methodological. Widely used in vitro assays do not measure the same chemical event, even when they are often grouped under the general label of “antioxidant activity”. The Folin–Ciocalteu (FC) response is often interpreted as a phenolic metric, yet in practice it mainly reflects reducing behavior and is not fully specific to phenolics. ABTS and DPPH both estimate radical scavenging, but they differ in steric accessibility, reaction medium, and kinetic sensitivity. Ferricyanide-based reducing power assays emphasize electron-transfer capacity rather than direct radical neutralization. Accordingly, current methodological reviews increasingly recommend complementary assay panels rather than single-endpoint interpretation [5,16,17]. One conceptual limitation common to all three radical-based endpoints used here should be stated explicitly: DPPH, ABTS·^+^, and the ferricyanide oxidant in the reducing-power assay are synthetic, non-physiological species that do not occur in biological systems. They therefore probe intrinsic reactivity toward stable artificial radicals rather than antioxidant efficacy under biological conditions, where superoxide, hydroxyl, and peroxyl radicals, along with peroxynitrite (a non-radical RNS), dominate —with additional factors such as compartmentalization, enzyme interplay, and metabolite chemistry also coming into play. For this reason, the present chemical rankings are explicitly intended as an early-stage comparative framework and not as a surrogate for physiologically relevant antioxidant performance. Recent phenolic-screening studies have also begun to pair antioxidant readouts with computational target-engagement or molecular docking assessment, reflecting a broader shift toward integrated early-stage prioritization rather than isolated chemical scoring [18].

Drug discovery—from hit identification through lead optimization to preclinical evaluation—relies on reliable early-stage chemical data. Antioxidant profiling of purified scaffolds serves a useful comparative function: it enables investigators to rank phenolic motifs by intrinsic redox reactivity, identify structural features associated with strong or weak performance, and establish a chemically grounded basis for further investigation. Phenolic frameworks continue to inspire medicinal chemistry programs because even modest structural modifications—such as the conversion of gallic acid to alkyl gallate esters, or the methylation of quercetin to improve metabolic stability—can shift the balance between potency, selectivity, and drug-likeness [19,20]. In that sense, a harmonized comparative dataset does not merely describe antioxidant capacity; it provides an actionable starting point for scaffold optimization, prodrug design, and formulation-oriented development within contemporary medicinal chemistry workflows.

At the same time, caution is required when translating chemical assay data into drug-discovery language. Strong performance in a cell-free assay does not automatically imply biological efficacy, target engagement, or favorable pharmacokinetics. The literature increasingly emphasizes an assay-to-biology gap: compounds that are highly reactive in chemical systems can differ markedly in stability, permeability, metabolism, plasma behavior, and cellular activity [5,6,21]. Nevertheless, standardized chemical profiling remains highly informative in the earliest stages of discovery because it allows investigators to identify lead-relevant motifs, reject weak scaffolds, and prioritize structures for further optimization, mechanistic study, or formulation work. In vitro antioxidant assays are not surrogates for preclinical validation, but they are useful filters for the rational triage of pharmaceutically relevant scaffolds.

**Scheme 1:**
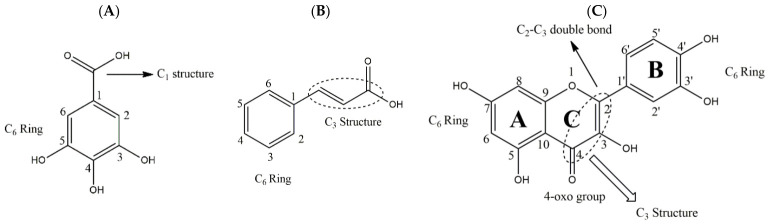
Representative structural frameworks of the three phenolic compound classes investigated in this study. (**A**) Gallic acid (3,4,5-trihydroxybenzoic acid), illustrating the C_6_–C_1_ hydroxybenzoic acid skeleton; (**B**) Cinnamic acid, illustrating the C_6_–C_3_ hydroxycinnamic acid skeleton; (**C**) Quercetin (3,3′,4′,5,7-pentahydroxyflavone), illustrating the C_6_–C_3_–C_6_ flavonoid skeleton with A-, B-, and C-ring labeling. Key structural features discussed in the text—including A-ring 5,7-dihydroxylation, B-ring 3′,4′-catechol motif, C-ring C_2_–C_3_ unsaturation, 4-oxo functionality, and 3-OH substitution—are indicated in panel (**C**). The structural formulae of all 21 tested compounds are presented in Scheme S1.

Against this background, the present study was designed as a side-by-side evaluation of major phenolic acids and flavonoids together with conventional synthetic comparators using Folin–Ciocalteu response, ABTS radical cation scavenging, DPPH radical scavenging, and reducing power assays under a unified analytical framework. The objective was not to make direct therapeutic claims, but to establish a robust comparative dataset from which chemically meaningful structure–activity relationships could be extracted. By examining hydroxybenzoic acids, hydroxycinnamic acids, flavonoids, a flavanone, and standard synthetic antioxidants in parallel, this study clarifies which structural patterns are associated with broad antioxidant performance, which are assay-selective, and which appear less promising as starting points for future medicinal-chemistry or formulation-oriented development. The representative structural frameworks of each compound class are presented in Scheme 1; the structural formulae of all 21 tested compounds are provided in the Supplementary Material (Scheme S1).

## 2. Results

The dataset showed pronounced assay-dependent variation, indicating that antioxidant performance depends on both scaffold class and specific substitution pattern (Table 1). No single structural rule explained all endpoints equally well; however, several recurring trends became clear when the assays were interpreted together.

### 2.1. Overall Comparative Antioxidant Profile

Among all tested compounds, gallic acid and quercetin emerged as the most consistently strong scaffolds across the assay panel. Gallic acid combined the highest Folin–Ciocalteu response (961.30 g GAE/kg), the strongest DPPH scavenging activity (IC_50_ = 4.45 µmol/L), and the highest reducing power (17.26 µmol TE/mg). Quercetin, in contrast, showed a more balanced profile, with high activity in ABTS (IC_50_ = 3.47 µmol/L), DPPH (IC_50_ = 5.20 µmol/L), and reducing power (12.78 µmol TE/mg). These two compounds therefore represent the clearest examples of broad-spectrum antioxidant performance in the present dataset.

**Figure 1:**
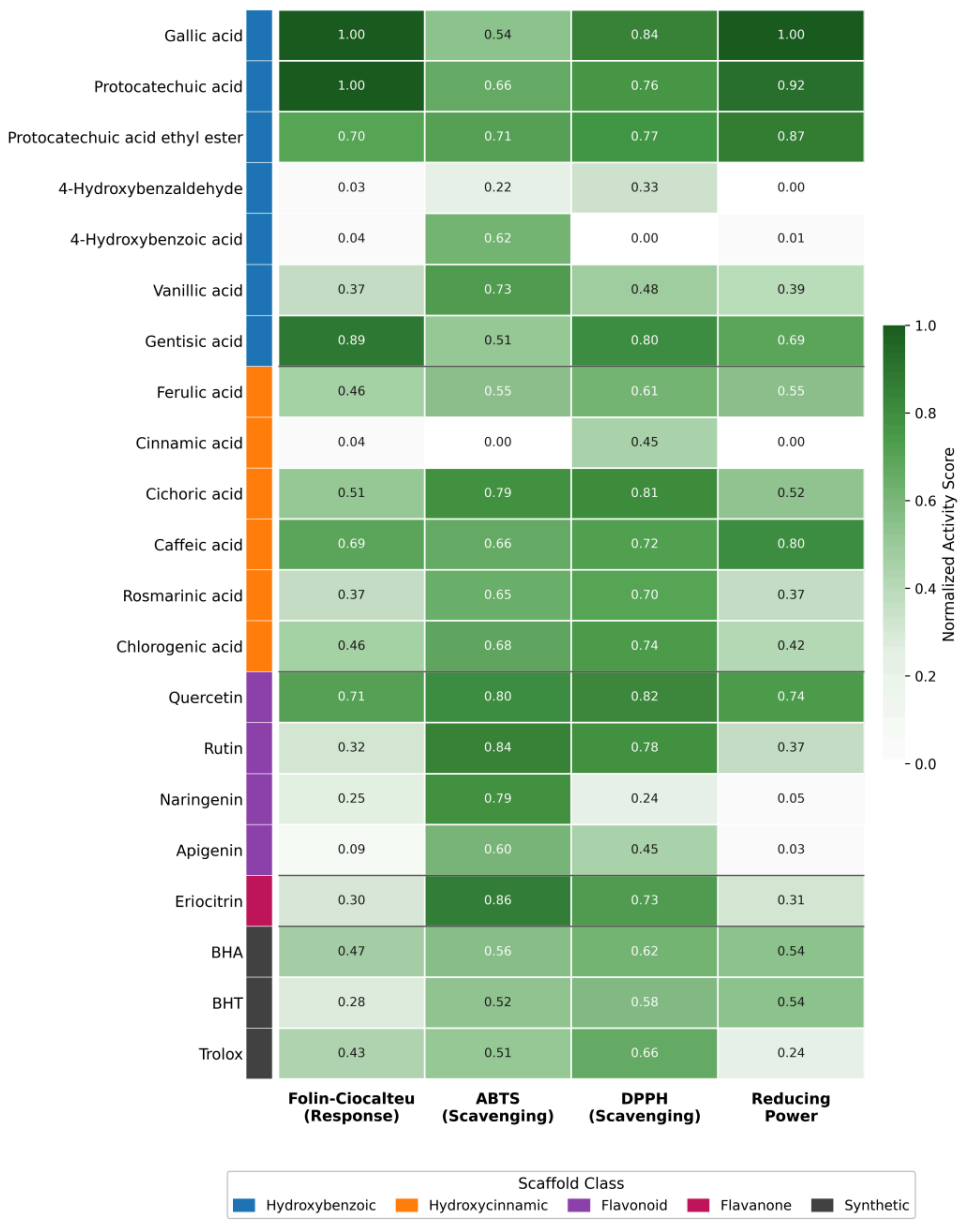
Heatmap of normalized antioxidant activity across four complementary in vitro assays. Activity scores are normalized to a 0–1 scale (higher = stronger). IC_50_-based assays were log_10_-transformed and inverted [1 − log_10_(IC_50_)/log_10_(max IC_50_)], so that higher scores consistently indicate stronger activity.

Other compounds showed strong but more assay-selective behavior. Eriocitrin, rutin, and cichoric acid stood out in the ABTS assay, whereas protocatechuic acid, protocatechuic acid ethyl ester, caffeic acid, and gentisic acid showed robust activity across multiple endpoints without matching the overall consistency of gallic acid or quercetin. At the other extreme, cinnamic acid, 4-hydroxybenzaldehyde, 4-hydroxybenzoic acid, and apigenin were weak in one or more assays, confirming that limited hydroxylation or unfavorable conjugation can markedly reduce antioxidant efficiency (Figure 1).

Relative to gallic acid, protocatechuic acid showed an almost equivalent FC response (99.61%), while quercetin (70.72%) and caffeic acid (68.98%) also responded strongly but less efficiently.

In the context of early-stage lead assessment, the potency of a compound is often categorized by its IC_50_ threshold. Generally, IC_50_ values below 10 µmol/L are considered highly potent for in vitro antioxidant assays. In the present study, gallic acid (4.45 µmol/L), quercetin (5.20 µmol/L), and cichoric acid (5.70 µmol/L) exhibited exceptional DPPH scavenging activity, falling well within this high-potency range. Compounds with IC_50_ values between 10 and 50 µmol/L, such as caffeic acid (12.93 µmol/L) and rosmarinic acid (15.58 µmol/L), represent robust antioxidant scaffolds with clear potential for structural optimization. In contrast, values exceeding 100 µmol/L, as seen with apigenin (159.14 µmol/L), indicate limited radical scavenging efficiency. 4-Hydroxybenzoic acid and naringenin did not reach 50% DPPH inhibition within the tested range and are therefore practically inactive under these conditions. Such scaffolds would require substantial structural modification to serve as effective redox-active starting points.

### 2.2. Hydroxybenzoic Acid Derivatives

The hydroxybenzoic acid subgroup demonstrated some of the clearest structure–activity contrasts in the entire study. Gallic acid, protocatechuic acid, and gentisic acid exhibited very high Folin–Ciocalteu responses and strong reducing power, indicating that dense hydroxylation strongly favors electron-donating behavior. Gallic acid was particularly dominant in DPPH scavenging, while protocatechuic acid and protocatechuic acid ethyl ester also showed strong DPPH activity. These results are consistent with the general chemical expectation that multiple hydroxyl substituents, especially ortho-related arrangements, facilitate both radical quenching and resonance stabilization of the resulting phenoxyl intermediates [11,14].

By contrast, mono-hydroxylated or weakly activated benzoic scaffolds performed poorly. 4-Hydroxybenzoic acid did not reach 50% DPPH inhibition within the tested concentration range, while 4-hydroxybenzaldehyde was similarly weak in both ABTS and DPPH and displayed reducing power below the detection limit of the assay (<0.01 µmol TE/mg), a value consistent with the absence of a sufficiently electron-rich aromatic system rather than a methodological artifact. Vanillic acid occupied an intermediate position: methoxyl substitution did not abolish antioxidant behavior, but the compound remained clearly less active than densely hydroxylated analogues. Taken together, the hydroxybenzoic acid series strongly supports the conclusion that hydroxylation density and substitution pattern are primary determinants of antioxidant potency in compact benzenoid scaffolds.

### 2.3. Hydroxycinnamic Acid Derivatives

The hydroxycinnamic acid group showed broader variation than the hydroxybenzoic acids, reflecting the influence of both phenolic substitution and side-chain conjugation. Cichoric acid was one of the strongest compounds in the entire study, with an ABTS IC_50_ of 3.63 µmol/L and a DPPH IC_50_ of 5.70 µmol/L, while still maintaining substantial reducing power. Caffeic acid also produced a strong and balanced profile, particularly in Folin–Ciocalteu response and reducing power. Chlorogenic acid and rosmarinic acid exhibited intermediate-to-strong activity, supporting the idea that conjugated hydroxycinnamic frameworks remain attractive redox-active scaffolds even when esterification or additional structural complexity is introduced.

Cinnamic acid itself, lacking hydroxyl substituents, was among the weakest compounds in the study. Its extremely poor ABTS and DPPH performance illustrates that conjugation alone is insufficient to drive potent antioxidant activity in the absence of appropriate electron-donating functionality. Ferulic acid, containing one methoxy and one hydroxyl substituent, showed intermediate performance and clearly trailed caffeic acid, which retains a more favorable hydroxylation pattern. This comparison suggests that the hydroxycinnamic scaffold is highly tunable: modest changes in substitution can shift the balance between radical scavenging and reducing behavior in a chemically interpretable manner.

### 2.4. Flavonoid and Synthetic Comparator Profiles

Flavonoids were especially prominent in the ABTS assay. Eriocitrin yielded the lowest ABTS IC_50_ value in the full dataset (2.47 µmol/L), followed closely by rutin, quercetin, cichoric acid, and naringenin. Quercetin also performed strongly in DPPH and reducing power, which is consistent with the recognized importance of the B-ring catechol system and conjugated flavonol framework [11]. Rutin retained excellent ABTS activity despite glycosylation, showing that glycosidic substitution does not necessarily eliminate strong cation-radical scavenging in aqueous systems. Eriocitrin showed a similar pattern, combining exceptional ABTS activity with moderate DPPH and reducing-power values.

Not all flavonoids were equally effective. Naringenin and apigenin showed marked assay selectivity, with relatively weak DPPH and reducing-power performance despite more favorable ABTS values. This divergence highlights that flavonoid class alone is not predictive; rather, hydroxyl distribution, conjugation, and glycosylation collectively shape behavior. Importantly, several natural phenolic compounds outperformed the synthetic comparators BHA, BHT, and Trolox in one or more assays. In ABTS scavenging, for example, quercetin, rutin, eriocitrin, naringenin, and cichoric acid all produced lower IC_50_ values than BHA and BHT. Such observations do not imply immediate pharmacological superiority, but they do establish that naturally occurring phenolic scaffolds can equal or exceed common synthetic reference antioxidants in controlled chemical systems.

The activity differences among the flavonoids reflect mainly the B-ring hydroxylation pattern, C-ring C2=C3 unsaturation, and the presence or absence of glycosylation; a detailed mechanistic interpretation of these structural contributions is provided in Discussion (Section 3.1).

A detailed scaffold-level structure–activity interpretation is provided in Supplementary Table S2.

## 3. Discussion

### 3.1. Assay-Dependent Structure–Activity Relationships

Across all four endpoints, the central message is not that a single compound is universally “best” but that different assays favor different structural features. The Folin–Ciocalteu and reducing-power assays broadly favored densely hydroxylated hydroxybenzoic acids and selected flavonoids, suggesting that these methods are strongly influenced by electron-transfer capacity. DPPH also rewarded hydroxyl-rich structures but discriminated more sharply against weakly substituted or poorly organized scaffolds. ABTS, in turn, highlighted several glycosylated and more structurally elaborate molecules, including eriocitrin and rutin, which were not the strongest reducing agents but were highly effective at cation-radical neutralization. This assay dependence is consistent with recent methodological analyses, which argue that antioxidant capacity cannot be captured by a single number without losing mechanistic meaning [5,16,17,22].

**Figure 2:**
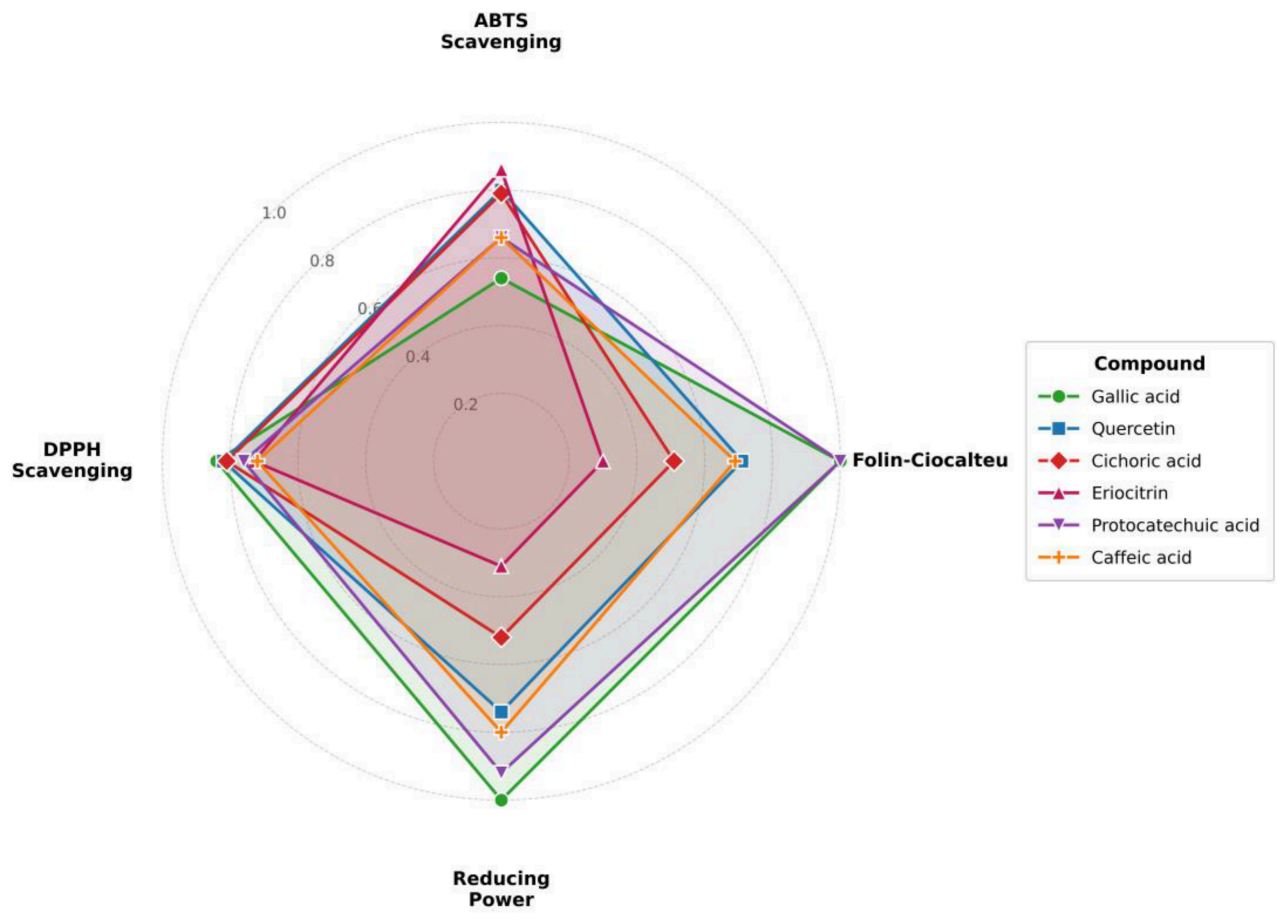
Radar plot of top-performing phenolic scaffolds across four antioxidant endpoints. Normalized activity scores (0–1) allow direct comparison of multi-assay profiles.

The present results therefore support a more nuanced use of antioxidant screening in chemistry-led projects. Rather than collapsing all outcomes into one rank order, investigators should distinguish between broad-spectrum redox-active scaffolds and compounds that excel only under specific assay conditions (Figure 2). From a structure–activity perspective, the most influential drivers in the current dataset were hydroxylation density, the presence of ortho-related phenolic groups, and extended conjugation. Glycosylation appeared to modify rather than abolish activity, especially in ABTS, suggesting that scaffold complexity and physicochemical context may shape accessibility to distinct radical systems.

The impact of glycosylation on antioxidant activity deserves closer examination across all four endpoints, not only ABTS. In the present dataset, the glycosylated flavonoids rutin and eriocitrin showed consistently lower Folin–Ciocalteu responses and reducing power than the aglycone quercetin, indicating that sugar conjugation reduces electron-transfer capacity. In the DPPH system, rutin (IC_50_ 7.57 µmol/L) and eriocitrin (IC_50_ 12.35 µmol/L) were moderately less active than quercetin (IC_50_ 5.20 µmol/L), suggesting partial steric shielding of the B-ring catechol. By contrast, the ABTS assay revealed an opposite trend: both glycosides outperformed quercetin in cation-radical neutralization (rutin IC_50_ 2.66 µmol/L; eriocitrin IC_50_ 2.47 µmol/L vs. quercetin IC_50_ 3.47 µmol/L). This discrepancy likely reflects the enhanced aqueous solubility conferred by the sugar moiety, which improves accessibility in the ABTS reaction medium. These findings are consistent with the broader literature, in which glycosylation effects on phenolic antioxidant activity remain contradictory. Several studies have reported that glycosylation diminishes radical scavenging by masking reactive hydroxyl groups and reducing planarity, whereas others have documented preserved or enhanced activity in aqueous-phase assays due to improved solubility and altered radical approach geometry. The present multi-assay dataset helps reconcile these apparently conflicting observations by demonstrating that glycosylation effects are assay-dependent rather than universally positive or negative: sugar conjugation penalizes electron-transfer and lipophilic radical scavenging endpoints while enhancing performance in aqueous cation-radical systems. Inter-assay correlation analysis (Table S1; Figure S2) further supported this view: Folin–Ciocalteu response and reducing power showed a strong positive correlation (Pearson r = 0.93, *p* < 0.001), consistent with their shared electron-transfer mechanism, whereas the Pearson correlation between DPPH and ABTS IC_50_ values was weak and non-significant (r = −0.05, *p* = 0.843), reflecting the distinct radical chemistries involved; however, the corresponding Spearman rank correlation was somewhat higher (ρ = 0.35), suggesting a modest monotonic trend obscured by outlier IC_50_ values.

A critical methodological finding of this study is that the Folin–Ciocalteu (FC) assay should not be interpreted as a precise or absolute measure of “total phenolic content.” Our data reveal that the FC response is governed more by the specific hydroxylation pattern and electron-transfer capacity of a molecule than by its mere identity as a phenolic compound. For instance, while gallic acid (a trihydroxybenzoic acid) defines the 100% reference point, structural analogs with lower hydroxyl density, such as 4-hydroxybenzoic acid and cinnamic acid, yielded negligible responses of only 3.87%. This discrepancy demonstrates that the FC reagent (phosphomolybdic/phosphotungstic acid) functions primarily as a non-specific oxidant for total reducing capacity rather than a selective probe for the phenolic functional group [23]. Consequently, relying solely on FC values can lead to a significant underestimation of total phenolics in samples dominated by monophenolic or weakly activated scaffolds, reinforcing the necessity of using a multi-assay battery for accurate scaffold assessment and early-stage lead evaluation [24].

These contrasts can be rationalized by examining the specific contributions of the flavonoid A-, B-, and C-ring systems (Scheme 1C). The superior performance of quercetin, rutin, and eriocitrin across multiple endpoints is primarily attributable to the 3′,4′-dihydroxy (catechol) arrangement on the B-ring, which facilitates hydrogen atom donation and stabilizes the resulting phenoxyl radical through intramolecular hydrogen bonding and extended resonance. In contrast, the 5,7-dihydroxy substitution pattern on the A-ring, present in both naringenin and apigenin, is insufficient on its own to drive strong DPPH or reducing-power activity when the B-ring carries only a single hydroxyl group; this limitation accounts for the pronounced assay selectivity observed with these two compounds. Equally important is the role of the C-ring: the C2=C3 double bond in quercetin (a flavonol) is conjugated with the 4-oxo group, creating an extended π-system that delocalizes the unpaired electron from the B-ring across the entire chromophore. Naringenin (a flavanone) lacks this C2=C3 unsaturation, which disrupts cross-ring conjugation and confines radical stabilization primarily to the B-ring. The additional 3-OH substituent on the C-ring of quercetin further enhances radical scavenging by providing a secondary site for hydrogen donation and by contributing to planarity-assisted resonance [11]. Notably, rutin retains exceptional ABTS activity despite glycosylation at the 3-OH position, indicating that sugar conjugation alters steric accessibility and physicochemical behavior without abolishing cation-radical neutralization capacity. Taken together, these observations establish a clear structural hierarchy for flavonoid antioxidant performance: B-ring catechol functionality is the primary determinant, C-ring C2=C3/4-oxo conjugation is a critical amplifier, and A-ring hydroxylation provides a supporting but insufficient contribution when acting alone.

### 3.2. Medicinal Chemistry Implications

The most useful interpretation of these data is not that the compounds are ready-made drug candidates, but that the assay-resolved structure–activity information identifies redox-active scaffolds that warrant differentiated follow-up in chemistry-oriented projects. The assay-wise interpretation of top-performing scaffolds is presented in Table 2, while the physicochemical properties and Lipinski drug-likeness criteria for all tested compounds are summarized in Table 3 to support this evaluation. Gallic acid and quercetin are high-value reference motifs because they combine strong activity with chemically well-defined substructures. Protocatechuic acid and caffeic acid also emerge as attractive minimalist scaffolds, showing that compact, synthetically accessible phenolic pharmacophores can retain meaningful redox activity. Cichoric acid, rutin, and eriocitrin are more structurally complex, yet their assay profiles indicate that scaffold expansion and glycosylation can preserve or even enhance certain forms of radical scavenging. Such molecules may therefore be especially relevant for prodrug design, nano-encapsulation, polymer conjugation, or self-emulsifying drug delivery systems (SEDDSs) rather than for direct potency-driven lead simplification. In particular, the multiple Lipinski violations and Veber TPSA exceedances observed for rutin and eriocitrin (Table 3) suggest that these glycosylated scaffolds are better positioned as substrates for formulation-driven development—where bioavailability can be engineered through carrier systems—than as conventional drug-like leads requiring oral absorption optimization. Among the minimalist scaffolds, protocatechuic acid is a particularly attractive candidate for prodrug design: its catechol motif and compact molecular framework (MW 154.12, cLogP 0.86) are amenable to ester or amide derivatization strategies aimed at improving lipophilicity and metabolic stability without introducing additional Lipinski violations. For the glycosylated scaffolds rutin and eriocitrin, whose multiple Lipinski violations preclude efficient passive oral absorption, recent advances in phenolic formulation offer viable delivery solutions. These include poly(lactic-co-glycolic acid) (PLGA) nanoparticles, SEDDSs, and liposomal encapsulation, all of which have been shown to enhance the oral bioavailability and plasma stability of polyphenolic compounds with high TPSA and low cLogP values [21].

At the same time, the present data should be interpreted with restraint. Chemical antioxidant assays do not establish target-based pharmacology, cellular protection, bioavailability, safety, or efficacy in vivo [5,6,21]. Polyphenols may suffer from poor permeability, rapid metabolism, limited stability [21,25], or nonspecific redox reactivity [26], all of which can complicate their progression within drug-discovery workflows. Indeed, many polyphenolic scaffolds are flagged as pan-assay interference compounds (PAINS) because of their tendency toward redox cycling, aggregation, and nonspecific protein binding, which can yield false positives in high-throughput screens. Within this dataset, catechol-bearing compounds—including protocatechuic acid, caffeic acid, quercetin, and their derivatives—carry the well-documented catechol PAINS alert, while the pyrogallol motif of gallic acid adds further liability through facile autoxidation and quinone formation. Compounds lacking these substructures, such as ferulic acid, apigenin, and naringenin, are less prone to PAINS-related interference, although their weaker antioxidant profiles may independently limit their utility as redox-active leads. This limitation underscores the importance of interpreting the present chemical data as preliminary scaffold rankings rather than validated hit confirmation.

A further consideration concerns the metabolic fate of polyphenols after oral administration. Dietary phenolic acids and flavonoids do not circulate as intact parent molecules; they undergo extensive Phase II conjugation (glucuronidation, sulfation, and methylation) in the small intestine and liver, as well as microbial transformation in the colon. As a result, the main species reaching plasma and peripheral tissues are typically glucuronide, sulfate, or methylated conjugates of the parent scaffolds, together with low-molecular-weight microbial metabolites such as protocatechuic acid, hydroxyphenylacetic acids, urolithins, and equol. These circulating metabolites often display markedly different radical-scavenging behavior from their precursors: conjugation of phenolic hydroxyl groups generally attenuates direct hydrogen-atom donation, while several metabolites retain bioactivity through non-antioxidant mechanisms, including Nrf2/ARE pathway activation, modulation of NF-κB signaling, and enzyme inhibition. Accordingly, the chemical rankings reported in this study should be interpreted as an intrinsic-reactivity profile of parent scaffolds rather than a predictor of in vivo antioxidant performance. Translation of these rankings to physiological relevance will require complementary assessment of (i) the principal circulating metabolites generated from each scaffold, (ii) their stability and reactivity under physiologically compatible conditions, and (iii) their activity in cellular or ex vivo models—for example, DCFH-DA-based cellular ROS assays, CAP-e (cell-based antioxidant protection of erythrocytes), ORAC with peroxyl-radical generation, and lipid-peroxidation inhibition in biologically relevant membranes. The harmonized parent-scaffold ranking provided here is therefore best regarded as a necessary chemical foundation on top of which such metabolite-aware and physiologically relevant assessments can be built.

Nevertheless, such limitations do not negate the value of the current screening approach. Instead, they define the next rational step: use assay-resolved structure–activity information to prioritize scaffolds for deeper characterization, including stability studies, computational property evaluation, mechanistic assays, and, where appropriate, cell-based validation. This integrated workflow is increasingly aligned with recent studies that couple antioxidant screening to molecular docking or computational property evaluation before making translational claims [18]. In that sense, the present work contributes a standardized benchmark for selecting phenolic templates of potential pharmaceutical interest while avoiding the overstatement that often surrounds extract-level antioxidant claims.

**Table 3:** Physicochemical properties and Lipinski drug-likeness assessment of tested phenolic compounds and synthetic comparators.

Compound	MW (g/mol)	cLogP	TPSA (Å^2^)	HBD	HBA	Lip. Viol.	RB	Drug-Likeness Assessment
Gallic acid	170.12	0.70	97.99	4	5	0	1	High polarity may limit passive permeation
Protocatechuic acid	154.12	0.86	77.76	3	4	0	1	Compact lead scaffold; no Lipinski violations
Protocatechuic acid ethyl ester	182.17	1.58	66.76	2	4	0	3	Improved lipophilicity vs. parent acid
4-Hydroxybenzaldehyde	122.12	1.35	37.30	1	2	0	1	Low MW fragment
4-Hydroxybenzoic acid	138.12	1.58	57.53	2	3	0	1	Minimal pharmacophore
Vanillic acid	168.15	1.43	66.76	2	4	0	2	Methoxy group improves lipophilicity
Gentisic acid	154.12	1.16	77.76	3	4	0	1	Compact lead scaffold
Ferulic acid	194.18	1.51	66.76	2	4	0	2	Favorable balance of properties
Cinnamic acid	148.16	2.13	37.30	1	2	0	2	Low MW; weak antioxidant
Cichoric acid	474.37	0.35	208.12	6	12	2	9	Two Lipinski violations (HBD, HBA); one Veber violation (TPSA = 208.12 > 140 Å^2^)
Caffeic acid	180.16	1.15	77.76	3	4	0	2	Strong candidate for ester derivatization
Rosmarinic acid	360.31	1.82	144.52	5	8	0	7	No Lipinski violations; TPSA exceeds Veber threshold (144.52 > 140 Å^2^), which may limit passive permeation
Chlorogenic acid	354.31	−0.36	164.75	6	9	1	5	One Lipinski violation (HBD); one Veber violation (TPSA = 164.75 > 140 Å^2^)
Quercetin	302.24	1.54	131.36	5	7	0	1	No Lipinski violations; TPSA borderline (131.36 Å^2^, approaching Veber threshold of 140 Å^2^)
Rutin	610.52	−0.87	269.43	10	16	3	6	Three Lipinski violations (MW, HBD, HBA); one Veber violation (TPSA = 269.43 > 140 Å^2^); delivery optimization required
Naringenin	272.25	2.52	86.99	3	5	0	1	Good drug-like profile
Apigenin	270.24	2.71	86.99	3	5	0	1	Weak antioxidant despite favorable properties
Eriocitrin	596.53	−0.73	245.29	9	15	3	7	Three Lipinski violations (MW, HBD, HBA); one Veber violation (TPSA = 245.29 > 140 Å^2^)
BHA	180.24	3.50	29.46	1	2	0	4	Highly lipophilic
BHT	220.35	5.10	20.23	1	1	1	2	cLogP > 5; excessive lipophilicity
Trolox	250.29	2.73	66.76	2	4	0	1	Balanced reference compound

MW: molecular weight; cLogP: calculated partition coefficient; TPSA: topological polar surface area; HBD: hydrogen bond donors; HBA: hydrogen bond acceptors; RB: rotatable bonds. Lipinski violations based on Lipinski’s Rule of Five (MW ≤ 500, cLogP ≤ 5, HBD ≤ 5, HBA ≤ 10) [19]. Veber oral bioavailability criteria: TPSA ≤ 140 Å^2^ and RB ≤ 10 [20]. Compounds exceeding either Veber threshold are flagged in the assessment column. The background colors in the table represent different chemical classes as follows: light blue for hydroxybenzoic acids, orange for hydroxycinnamic acids, purple for flavonoids, green for flavanones, and grey/white for synthetic comparators (BHA, BHT, and Trolox). All physicochemical descriptors were retrieved from PubChem (https://pubchem.ncbi.nlm.nih.gov (accessed on 1 March 2026)) and cross-verified using SwissADME [27]; http://swissadme.ch (accessed on 1 March 2026).

## 4. Materials and Methods

### 4.1. Chemicals and Reference Compounds

The comparative profiling study was conducted using a panel of high-purity phytochemical entities and synthetic comparators. The hydroxybenzoic acid group comprised gallic acid (G7384, ≥97.5%), protocatechuic acid (P5630, ≥97%), protocatechuic acid ethyl ester (E24859, ≥97%), 4-hydroxybenzaldehyde (144088, ≥98%), 4-hydroxybenzoic acid (H5376, ≥99%), vanillic acid (H36001, ≥97%), and gentisic acid (G5127, ≥98%), all obtained from Sigma-Aldrich (St. Louis, MO, USA). The hydroxycinnamic acid derivatives included ferulic acid (128708, ≥99%), cinnamic acid (C80857, ≥99%), cichoric acid (C7243, ≥95%), caffeic acid (C0625, ≥98%), rosmarinic acid (R4033, ≥98%), and chlorogenic acid (C3878, ≥95%); the flavonoid set comprised quercetin (Q4951, ≥95%), rutin (R5143, ≥94%), naringenin (N5893, ≥95%), apigenin (A3145, ≥97%), and eriocitrin (SMB00349, ≥95%), all sourced from Sigma-Aldrich. Synthetic reference antioxidants, specifically butylated hydroxyanisole (BHA; B1253, ≥98.5%), butylated hydroxytoluene (BHT; W218405, ≥99%), and Trolox (238813, ≥97%), were acquired from Merck (Darmstadt, Germany). For the in vitro assays, all specialized reagents were of analytical grade and used as received without further purification. These included Folin–Ciocalteu’s phenol reagent, sodium carbonate (Na_2_CO_3_), 2,2′-azino-bis(3-ethylbenzothiazoline-6-sulfonic acid) (ABTS), potassium persulfate (K_2_S_2_O_8_), 2,2-diphenyl-1-picrylhydrazyl (DPPH˙), potassium ferricyanide (K_3_Fe(CN)_6_), trichloroacetic acid (TCA), and ferric chloride (FeCl_3_), all of which were supplied by Sigma-Aldrich (St. Louis, MO, USA) and Merck (Darmstadt, Germany). High-purity ethanol, distilled water (obtained from a Milli-Q water purification system, Merck Millipore, Darmstadt, Germany), and phosphate buffer salts used for solution preparation and pH adjustment were also of analytical grade.

### 4.2. Preparation of Standard Solutions

Stock solutions of each tested compound were prepared at a concentration of 1 mg/mL in ethanol (for DPPH and reducing power assays) or in phosphate buffer (for ABTS and Folin–Ciocalteu assays); compounds with limited aqueous solubility (quercetin, apigenin, naringenin) were first dissolved in a minimal volume of dimethyl sulfoxide (DMSO, ≤0.5% *v*/*v* final concentration) before dilution. Solvent controls confirmed that residual DMSO at this level did not interfere with any assay readout. These stock solutions were used in all subsequent assays, including Folin–Ciocalteu response, ABTS radical cation scavenging, DPPH radical scavenging, and reducing power evaluation. Aliquot volumes for each assay were selected according to the protocol-specific working range needed for concentration–response analysis. All measurements were performed in triplicate.

### 4.3. Folin–Ciocalteu Response

The Folin–Ciocalteu response of the tested compounds was determined as previously described [28]. Briefly, 100 µL of stock solution was mixed with 4.5 mL of distilled water, followed by the addition of 100 µL of Folin–Ciocalteu reagent. After 3 min, 300 µL of 2% Na_2_CO_3_ solution was added. The mixture was vortexed and incubated at room temperature for 2 h, and absorbance was then recorded at 760 nm against the blank. Quantification was performed using a gallic acid calibration curve. Results were expressed as g gallic acid equivalents per kg of tested compound (g GAE/kg). The gallic acid calibration curve is provided in Figure S1.

### 4.4. ABTS Radical Cation Scavenging Assay

ABTS radical cation (ABTS^+^) scavenging activity was determined as previously described [29]. A 0.1 M phosphate buffer (pH 7.4), 2 mM ABTS solution, and 2.45 mM K_2_S_2_O_8_ solution were prepared. The ABTS and K_2_S_2_O_8_ solutions were mixed at a 1:2 ratio and kept in the dark for 6 h to generate the ABTS radical cation; complete radical formation was verified by monitoring absorbance stability at 734 nm prior to use. Aliquots of stock solutions (5, 10, 20, 40, 80, and 160 µL) were transferred into test tubes, 1 mL of ABTS/K_2_S_2_O_8_ working solution was added, and the final volume was adjusted to 4 mL with phosphate buffer. After 30 min of incubation at room temperature, absorbance was recorded at 734 nm. Radical scavenging activity was calculated as percentage inhibition, and IC_50_ values were obtained from concentration–response plots and expressed as µmol/L.

### 4.5. DPPH Radical Scavenging Assay

DPPH radical scavenging activity was evaluated as previously described [30]. Aliquots of stock solutions (2.5, 5, 10, 20, 40, and 80 µL) were transferred to test tubes and brought to 3 mL with ethanol. Subsequently, 1 mL of 0.26 mM DPPH solution in ethanol was added, and the mixture was vortexed. The reaction mixtures were incubated at room temperature in the dark for 30 min, after which absorbance was measured at 517 nm. Radical scavenging activity was calculated as percentage inhibition, and IC_50_ values were derived from concentration–response plots and expressed as µmol/L.

### 4.6. Reducing Power Assay

Reducing power was determined using a previously described method with minor modifications [31,32]. Aliquots of stock solutions (5, 10, 20, and 40 µL) were transferred into test tubes and adjusted to 1.25 mL with 0.2 M phosphate buffer (pH 6.6). Then, 1.25 mL of 1% K_3_Fe(CN)_6_ solution was added. The mixtures were incubated at 50 °C for 20 min. Following incubation, 1.25 mL of 10% trichloroacetic acid and 0.25 mL of 0.1% FeCl_3_ solution were added, the mixtures were vortexed again, and absorbance was recorded at 700 nm. Results were expressed as µmol Trolox per mg of compound (µmol TE/mg).

### 4.7. Data Handling and Comparative Interpretation

Folin–Ciocalteu results were expressed as g GAE/kg. The Folin–Ciocalteu response of gallic acid corresponded to 961.30 g GAE/kg, representing 96.13% of the theoretical maximum (1000 g GAE/kg), which confirms acceptable analytical accuracy for the calibration system. To facilitate systematic comparison, other entities were ranked by their Relative FC Response (% of gallic acid), calculated by normalizing each compound’s g GAE/kg value against gallic acid (set at 100.00%). Because this ratio is derived from mean values, its uncertainty is implicit in the SD of the parent g GAE/kg measurement. Radical scavenging activities for ABTS and DPPH assays were calculated as percentage inhibition using the following formula:

$$Inhibition\left(\%\right)=\left[\frac{A_{blank}-A_{sample}}{A_{blank}}\right]\times 100$$

IC_50_ values, representing the concentration of the compound required to inhibit 50% of the radical activity, were determined by fitting a four-parameter logistic (4PL) sigmoidal model (top constrained to 100%, bottom to 0%, Hill slope unconstrained) to the concentration–response data using GraphPad Prism 9.0. For compounds whose IC_50_ fell outside the directly measured concentration window (e.g., 4-hydroxybenzoic acid in DPPH, naringenin in DPPH), values were obtained by model extrapolation and should therefore be interpreted as approximate estimates rather than precisely determined endpoints; these cases are noted in the text. Representative concentration–response curves are shown in Figure S3. Reducing power, which is an absorbance-based endpoint without an inhibition curve, was expressed as µmol TE/mg calculated from a Trolox calibration curve; for this assay, group differences were assessed by one-way ANOVA rather than non-linear regression. All experiments were performed in triplicate (*n* = 3), and the results are presented as mean ± standard deviation.

### 4.8. Statistical Analysis

All experimental measurements were performed in triplicate (*n* = 3) to ensure reproducibility, and the resulting data are expressed as mean ± standard deviation (SD). Statistical significance and the distribution of data were evaluated using one-way analysis of variance (ANOVA), followed by post hoc tests where appropriate to determine differences between the tested compounds. IC_50_ values and their associated 95% confidence intervals were derived from non-linear regression models (log[inhibitor] vs. normalized response). All statistical calculations and curve fitting were performed using GraphPad Prism 9.0 (GraphPad Software, San Diego, CA, USA). Each measurement was performed in triplicate (*n* = 3) from independently weighed aliquots; however, all replicates were prepared from the same batch of reagents and analyzed within a single analytical session. Accordingly, the reported variability reflects within-day analytical precision (technical replication) rather than between-day or between-batch reproducibility. A *p*-value of <0.05 was considered statistically significant.

## 5. Conclusions

This study provides a systematic comparative profile of major phenolic acids, flavonoids, a flavanone, and common synthetic antioxidants across four complementary in vitro assays. The data show that antioxidant behavior is strongly assay-dependent and closely tied to scaffold architecture. Gallic acid dominated DPPH scavenging and reducing power, whereas quercetin combined high activity across ABTS, DPPH, and reducing power, placing it among the most consistently active compounds in the dataset. Eriocitrin and rutin were especially effective in the ABTS system, while cichoric acid, protocatechuic acid, protocatechuic acid ethyl ester, caffeic acid, and gentisic acid also displayed strong multi-assay performance. The study also confirms that the Folin–Ciocalteu assay measures total reducing capacity rather than a specific phenolic count, as shown by the minimal response of weakly or non-hydroxylated scaffolds such as 4-hydroxybenzoic acid and cinnamic acid (3.87% relative to gallic acid); this finding reinforces the necessity of multi-assay profiling for reliable scaffold evaluation. The dataset, however, characterizes the intrinsic chemical reactivity of parent scaffolds toward synthetic radicals and should not be equated with antioxidant efficacy under physiological conditions, where metabolic conjugation, microbial biotransformation, and tissue compartmentalization substantially reshape the species actually reaching biological targets.

From a broader chemical standpoint, the results confirm that hydroxylation density, ortho-related substitution, and extended conjugation are key drivers of antioxidant performance, whereas weakly substituted or poorly activated aromatic systems are much less effective. For medicinal chemistry and early-stage lead assessment, this dataset is valuable because it distinguishes broad-spectrum redox-active scaffolds from assay-selective ones and provides a rational basis for scaffold triage. Future work should focus on translating these chemical findings into biologically informative models, including stability assessment, bioavailability-oriented optimization, and cell-based validation, so that lead-relevant phenolic scaffolds can be evaluated more realistically within drug-discovery pipelines.

The physicochemical property data (Table 3) complement the antioxidant profiles by highlighting structural features relevant to bioavailability. Compact scaffolds such as gallic acid and protocatechuic acid satisfy both Lipinski and Veber criteria, indicating favorable drug-like profiles for passive oral absorption, whereas structurally complex compounds such as rutin and eriocitrin carry multiple Lipinski violations that may limit passive absorption. These observations do not constitute translational recommendations, but they may help inform the design of future studies that incorporate stability testing, permeability assessment, or formulation strategies [21,25]. Together with established drug-likeness criteria [19,20], these property data provide a complementary filter for ADME-oriented candidate selection and, where appropriate, target-based follow-up [18]. By coupling assay-resolved antioxidant data with property assessment, this work offers a structured comparative foundation that bridges phytochemical profiling and chemistry-guided prioritization.

The principal contribution of this work lies in the harmonized comparison of 21 structurally diverse phenolic compounds across four complementary assay systems under identical analytical conditions. While individual antioxidant data for many of these compounds exist in the literature, they are typically generated under heterogeneous protocols that preclude direct cross-study ranking. The present dataset addresses this gap by providing internally consistent, assay-resolved structure–activity information within a single experimental framework.

Several limitations should be acknowledged. First, the study is restricted to cell-free chemical assays and does not include cellular, ex vivo, or in vivo validation; accordingly, the reported rankings reflect intrinsic chemical reactivity rather than biological antioxidant efficacy. Second, all measurements were performed as technical triplicates within a single analytical session, so the reported precision represents within-day repeatability and not full experimental reproducibility. Third, the panel of synthetic comparators (BHA, BHT, Trolox) was selected for methodological consistency with established antioxidant literature but does not encompass all widely studied reference antioxidants such as ascorbic acid or curcumin derivatives.

Future investigations should pursue four complementary directions: (i) evaluation of the top-ranked scaffolds using biologically relevant assays such as ORAC, cellular ROS scavenging, and lipid peroxidation inhibition; (ii) integrated metabolite-aware evaluation combining assessment of Phase I/II biotransformation profiles (glucuronide, sulfate, methylated conjugates) and microbial metabolites (e.g., urolithins, hydroxyphenylacetic acids, equol) with physiologically relevant antioxidant assays such as DCFH-DA-based cellular ROS measurement, CAP-e, peroxyl-radical-based ORAC, and lipid-peroxidation inhibition, in order to determine whether the chemical rankings reported here translate into biologically meaningful antioxidant behavior; (iii) computational validation through DFT-derived bond dissociation enthalpies and ionization potentials to provide mechanistic support for the empirical rankings; and (iv) formulation-oriented studies (e.g., PLGA nanoparticles, SEDDSs, liposomal encapsulation) for glycosylated scaffolds whose Lipinski violations preclude efficient passive absorption.

## Figures and Tables

**Table 1:** Comprehensive antioxidant profile of phenolic compounds and synthetic comparators.

Category	Compound	Folin–Ciocalteu (g GAE/kg)	Relative FC Response (% of Gallic Acid)	ABTS IC_50_ (µmol/L)	DPPH IC_50_ (µmol/L)	Reducing Power (µmol TE/mg)
Hydroxybenzoic acids	Gallic acid	961.30 ± 4.2 ^a^	100.00	17.57 ± 0.84 ^c^	4.45 ± 0.22 ^d^	17.26 ± 0.52 ^a^
	Protocatechuic acid	957.59 ± 3.8 ^a^	99.61	8.31 ± 0.42 ^d^	9.04 ± 0.45 ^d^	15.85 ± 0.48 ^b^
	Protocatechuic acid ethyl ester	673.74 ± 4.9 ^c^	70.09	6.13 ± 0.31 ^d^	8.38 ± 0.42 ^d^	15.05 ± 0.45 ^b^
	4-Hydroxybenzaldehyde	24.11 ± 0.6 ^p^	2.51	134.28 ± 6.71 ^b^	480.60 ± 24.03 ^c^	<0.01 ^j^
	4-Hydroxybenzoic acid	37.22 ± 0.8 ^o^	3.87	11.11 ± 0.56 ^c^	>1000 ^a^	0.15 ± 0.01 ^i^
	Vanillic acid	359.44 ± 2.8 ^i^	37.39	5.46 ± 0.27 ^d^	121.03 ± 6.05 ^c^	6.80 ± 0.20 ^f^
	Gentisic acid	856.33 ± 5.1 ^b^	89.08	21.08 ± 1.05 ^c^	6.10 ± 0.31 ^d^	11.89 ± 0.36 ^d^
Hydroxycinnamic acids	Ferulic acid	440.93 ± 4.1 ^g^	45.87	16.89 ± 0.85 ^c^	36.72 ± 1.84 ^d^	9.42 ± 0.28 ^e^
	Cinnamic acid	37.22 ± 0.9 ^o^	3.87	>200 ^a^	159.55 ± 7.98 ^c^	0.07 ± 0.01 ^j^
	Cichoric acid	486.33 ± 3.2 ^e^	50.59	3.63 ± 0.18 ^d^	5.70 ± 0.29 ^d^	9.00 ± 0.27 ^e^
	Caffeic acid	663.15 ± 5.5 ^d^	68.98	8.46 ± 0.42 ^d^	12.93 ± 0.65 ^d^	13.81 ± 0.41 ^c^
	Rosmarinic acid	351.52 ± 3.1 ^i^	36.57	8.86 ± 0.44 ^d^	15.58 ± 0.78 ^d^	6.44 ± 0.19 ^f^
	Chlorogenic acid	442.78 ± 3.7 ^g^	46.06	7.42 ± 0.37 ^d^	10.98 ± 0.55 ^d^	7.23 ± 0.22 ^f^
Flavonoids	Quercetin	679.82 ± 6.4 ^c^	70.72	3.47 ± 0.17 ^d^	5.20 ± 0.26 ^d^	12.78 ± 0.38 ^d^
	Rutin	303.89 ± 4.4 ^j^	31.61	2.66 ± 0.13 ^d^	7.57 ± 0.38 ^d^	6.33 ± 0.19 ^f^
	Naringenin	240.93 ± 3.6 ^m^	25.06	3.75 ± 0.19 ^d^	>500 ^a^	0.89 ± 0.03 ^i^
	Apigenin	87.07 ± 1.5 ^n^	9.06	12.12 ± 0.61 ^c^	159.14 ± 7.96 ^c^	0.44 ± 0.01 ^i^
Flavanone	Eriocitrin	285.45 ± 2.8 ^k^	29.69	2.47 ± 0.12 ^d^	12.35 ± 0.62 ^d^	5.35 ± 0.16 ^g^
Synthetic comparators	BHA	455.74 ± 2.9 ^f^	47.41	15.40 ± 0.77 ^c^	32.62 ± 1.63 ^d^	9.30 ± 0.28 ^e^
	BHT	270.56 ± 2.2 ^l^	28.14	19.68 ± 0.98 ^c^	49.21 ± 2.46 ^d^	9.27 ± 0.28 ^e^
	Trolox	411.30 ± 3.5 ^h^	42.79	21.04 ± 1.05 ^c^	22.50 ± 1.13 ^d^	4.11 ± 0.12 ^h^

Lower IC_50_ values indicate stronger radical scavenging activity; higher reducing-power values indicate stronger electron-transfer capacity. All values are expressed as mean ± SD (*n* = 3). One-way ANOVA confirmed statistically significant differences among compounds for each assay endpoint (*p* < 0.001). Pairwise comparisons (Tukey’s HSD, *p* < 0.05) were performed using GraphPad Prism 9.0; grouping letters indicating statistically homogeneous subsets are presented alongside the mean values in this table. Means within each column sharing the same superscript letter are not significantly different (*p* > 0.05). The high F-statistics reflect the low within-group variance characteristic of technical replication from a single analytical session; the reported precision should therefore be interpreted as analytical repeatability rather than full experimental reproducibility. IC_50_ exceeded the directly measured concentration range; the compound is considered practically inactive under the present assay conditions. Values are not reported numerically to avoid false precision from model extrapolation.

**Table 2:** Assay-wise interpretation of top-performing compounds.

Assay	Top-Performing Compounds	Dominant Chemical Emphasis	Interpretive Takeaway
Folin–Ciocalteu response	Gallic acid, protocatechuic acid, gentisic acid	Overall reducing response/electron-donating capacity	Highly hydroxylated hydroxybenzoic acids dominate this endpoint [5,7,16]
ABTS radical cation scavenging	Eriocitrin, rutin, quercetin, cichoric acid, naringenin	Cation-radical neutralization in aqueous buffer	This assay favors several flavonoid and polyfunctional hydroxycinnamic scaffolds [5,10,12]
DPPH radical scavenging	Gallic acid, quercetin, cichoric acid, gentisic acid	Hydrogen donation to a stable organic radical	Hydroxyl density and radical stabilization dominate rank order [8,9]
Reducing power	Gallic acid, protocatechuic acid, protocatechuic acid ethyl ester, caffeic acid, quercetin	Electron-transfer capacity toward the ferricyanide system	Pattern broadly parallels Folin–Ciocalteu response [5,7]

## Data Availability

All data generated during this study are included in the published article and its Supplementary Materials.

## Supplementary Materials

The following supporting information can be downloaded at: https://www.mdpi.com/article/10.3390/molecules31091478/s1. Scheme S1: Structural formulae of all phenolic compounds and synthetic comparators tested in this study; Table S1: Inter-assay correlation matrix (Pearson r/Spearman ρ); Table S2: Scaffold-level structure–activity interpretation; Figure S1: Gallic acid calibration curve; Figure S2: Inter-assay correlation scatter matrix; Figure S3: Representative concentration–response curves for DPPH and ABTS assays with 4PL model fits and R^2^ values.

## Abbreviations

The following abbreviations are used in this manuscript:

4PL	Four-parameter logistic
ABTS	2,2′-Azino-bis(3-ethylbenzothiazoline-6-sulfonic acid)
ADME	Absorption, distribution, metabolism, excretion
ANOVA	Analysis of variance
ARE	Antioxidant response element
BHA	Butylated hydroxyanisole
BHT	Butylated hydroxytoluene
CAP-e	Cell-based antioxidant protection in erythrocytes
cLogP	Calculated partition coefficient
DCFH-DA	2′,7′-Dichlorodihydrofluorescein diacetate
DFT	Density functional theory
DMSO	Dimethyl sulfoxide
DPPH	2,2-Diphenyl-1-picrylhydrazyl
FC	Folin–Ciocalteu
FRAP	Ferric reducing antioxidant power
GAE	Gallic acid equivalents
HAT	Hydrogen atom transfer
HBA	Hydrogen bond acceptors
HBD	Hydrogen bond donors
HSD	Honestly significant difference
IC_50_	Half-maximal inhibitory concentration
MW	Molecular weight
NF-κB	Nuclear factor-kappa-light-chain-enhancer of activated B-cells
Nrf2	Nuclear factor erythroid 2-related factor 2
ORAC	Oxygen radical absorbance capacity
PAINS	Pan-assay interference compounds
PLGA	Poly(lactic-co-glycolic acid)
RB	Rotatable bonds
ROS	Reactive oxygen species
RP	Reducing power
SAR	Structure–activity relationship
SD	Standard deviation
SEDDS	Self-emulsifying drug delivery system
SET	Single-electron transfer
TCA	Trichloroacetic acid
TE	Trolox equivalents
TPSA	Topological polar surface area
