# Supplementary material for: Comparative Antioxidant Profiling of Phenolic Acids and Flavonoids: Assay-Resolved Structure–Activity Relationships Under Harmonized In Vitro Conditions
Source: Molecules. 2026 Apr 29;31(9):1478. doi: 10.3390/molecules31091478 (PMC13165421; doi:10.3390/molecules31091478)
Supplement: Supplementary file 1 [file molecules-31-01478-s001.zip › molecules-4248170-supplementary.pdf]

Supplementary Material

Comparative Antioxidant Profiling of Phenolic Acids and Flavonoids: Assay-Resolved Structure–Activity Relationships Under Harmonized *In Vitro* Conditions

Category	Compound	Chemical Structure
Hydroxybenzoic acids	Gallic acid	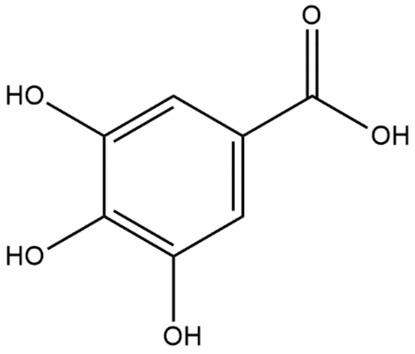
	Protocatechuic acid	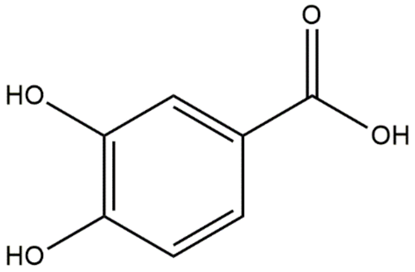
	Protocatechuic acid ethyl ester	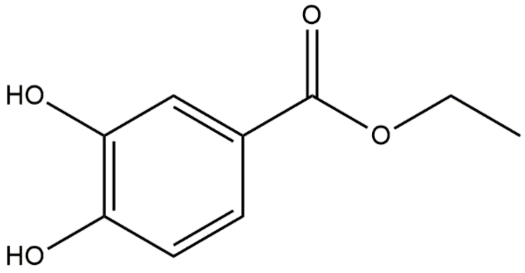
	4-Hydroxybenzaldehyde	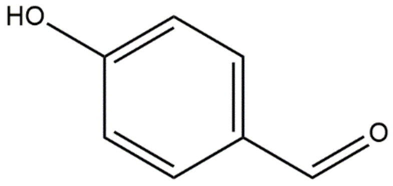
	4-Hydroxybenzoic acid	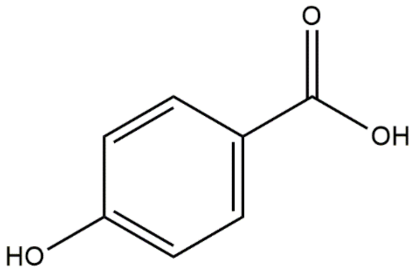

	Vanillic acid	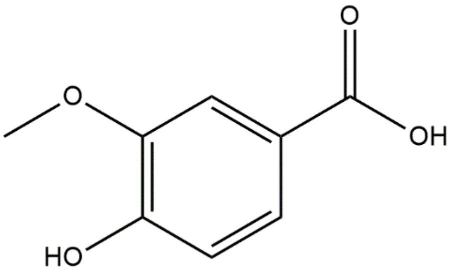
	Gentisic acid	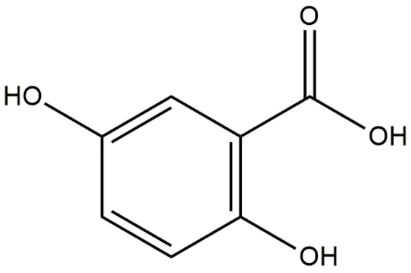
Hydroxycinnamic acids	Ferulic acid	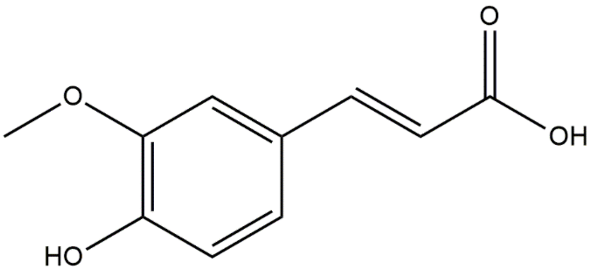
	Cinnamic acid	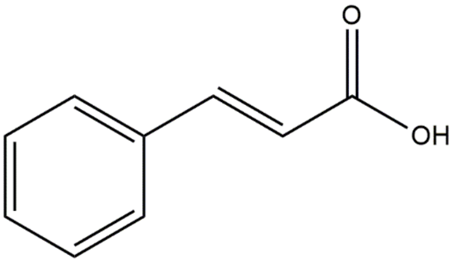
	Cichoric acid	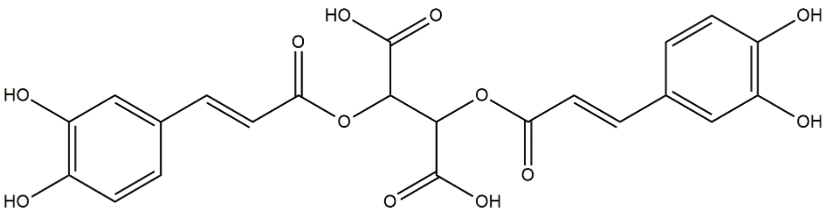
	Caffeic acid	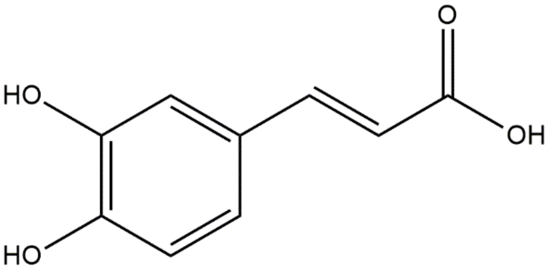

	Rosmarinic acid	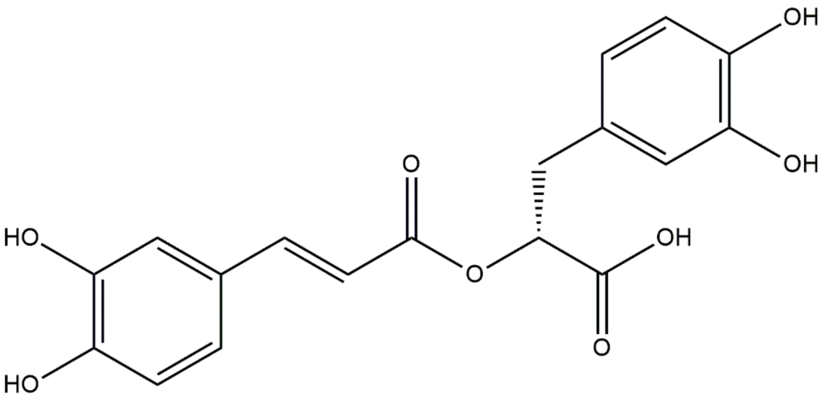
	Chlorogenic acid	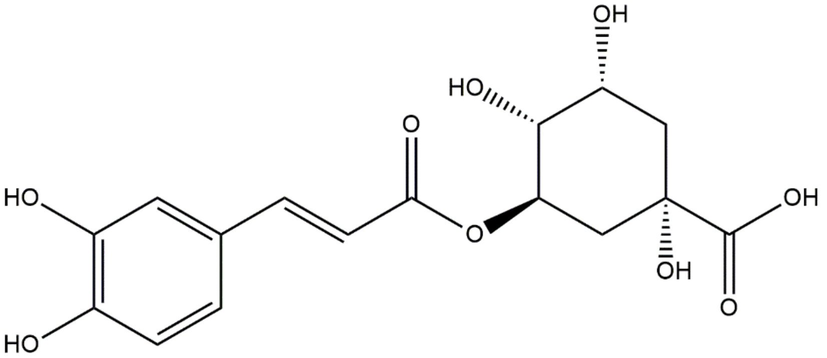
Flavonoids	Quercetin	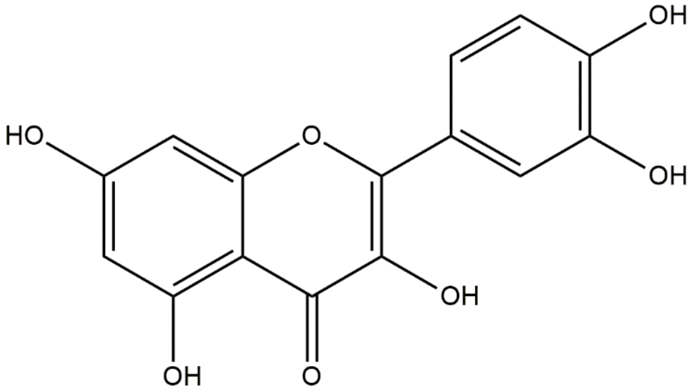

Flavanone

	Rutin	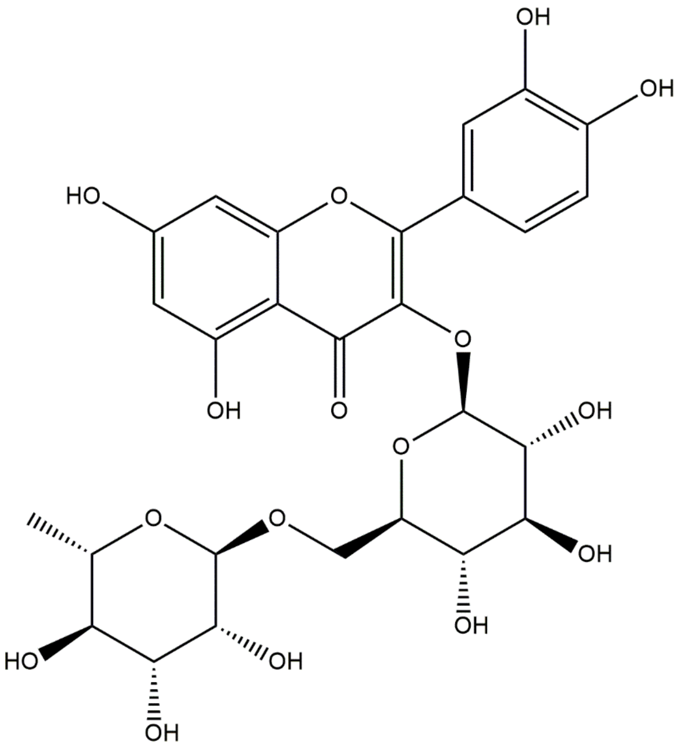
	Naringenin	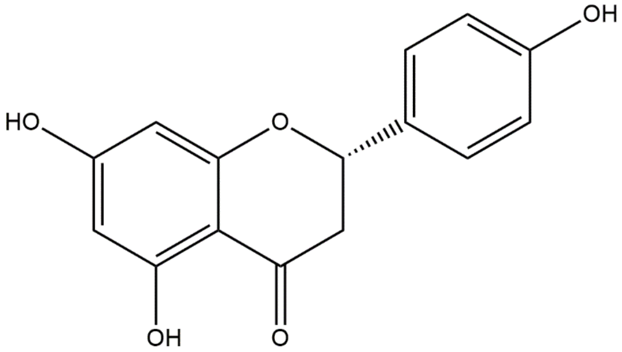
	Apigenin	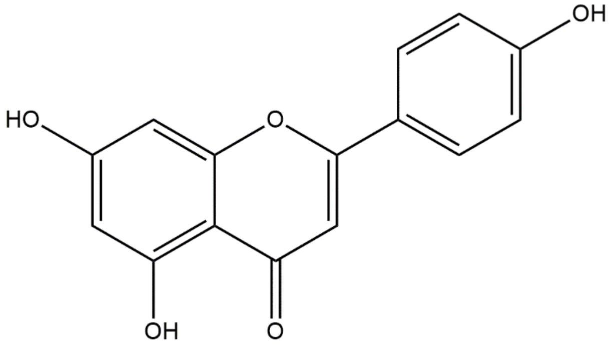
Flavanone	Eriocitrin	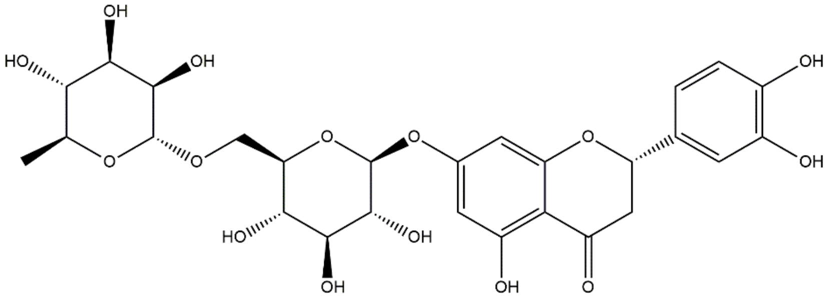

Synthetic comparators	BHA	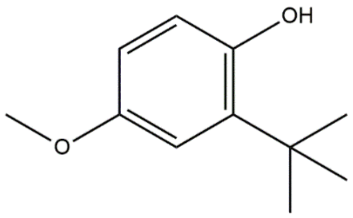
	BHT	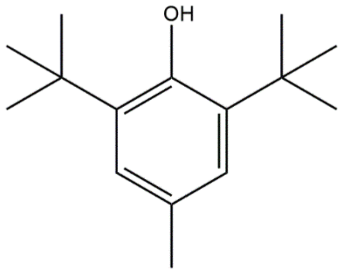
	Trolox	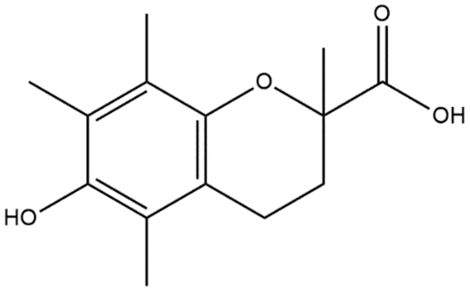

Scheme S1. Structural formulae of all phenolic compounds and synthetic comparators tested in this study, grouped by compound class: hydroxybenzoic acids, hydroxycinnamic acids, flavonoids, a flavanone, and synthetic comparators.

Table S1. Inter-assay correlation matrix. Pearson r values are shown below the diagonal (roman); Spearman ρ values are shown above the diagonal (italic).

	**FC (g GAE/kg)**	**ABTS IC_{50} (µmol/L)**	**DPPH IC_{50} (µmol/L)**	**RP (µmol TE/mg)**
FC	**1.00**	*-0.17* ^{ns}	*-0.83* ***	*+0.93* ***
ABTS	-0.38 ^{ns}	**1.00**	*+0.35* ^{ns}	*-0.15* ^{ns}
DPPH	-0.36 ^{ns}	-0.05 ^{ns}	**1.00**	*-0.72* ***
RP	+0.93 ***	-0.38 ^{ns}	-0.37 ^{ns}	**1.00**

**** $p < 0.001$; ** $p < 0.01$; * $p < 0.05$; ns = not significant. n = 21 compounds. Correlations were computed on raw (untransformed) assay values.*

Note: *FC and reducing power are directly measured response variables (higher = more active); ABTS and DPPH are IC_{50} values (lower = more active). Consequently, negative correlations between FC/RP and IC_{50}-based assays indicate concordant antioxidant activity.*

Interpretation: *The strong positive correlation between FC and reducing power (Pearson $r = +0.93$, $p < 0.001$; Spearman $\rho = +0.93$, $p < 0.001$) is consistent with their shared electron-transfer mechanism. The correlation between DPPH and ABTS IC_{50} values was weak and non-significant (Pearson $r = -0.05$, $p = 0.843$; Spearman $\rho = +0.35$, $p = 0.122$), reflecting the distinct radical chemistries and steric requirements of each system. The strong negative Spearman correlation between FC and DPPH IC_{50} ($\rho = -0.83$, $p < 0.001$) indicates that compounds with high electron-donating capacity also tend to be potent hydrogen-atom donors, although this rank-order relationship is attenuated by outlier IC_{50} values in the Pearson analysis ($r = -0.36$, $p = 0.109$).*

Table S2. Structure–activity interpretation of the major scaffold classes.

Structural feature	Representative compounds	Typical assay behavior	Chemical interpretation	Physicochemical note
Trihydroxy benzoic scaffold	Gallic acid	Very high Folin–Ciocalteu response, strongest DPPH scavenging, highest reducing power	Dense hydroxylation promotes electron donation and radical stabilization	Useful redox-active template for lead-inspired scaffold design; polarity and metabolic liability should be considered
Ortho-dihydroxy benzoic scaffold	Protocatechuic acid, gentisic acid	Consistently strong multi-assay activity, especially DPPH and reducing power	Catechol-like arrangement supports resonance-assisted quenching and reducing capacity	Lead-relevant benchmark for compact phenolic pharmacophores
Mono-hydroxylated benzoic scaffold	4-Hydroxybenzoic acid, 4-hydroxybenzaldehyde	Weak across most assays; extremely poor DPPH performance	Insufficient hydroxylation limits hydrogen donation and electron transfer	Low priority as standalone antioxidant scaffolds unless further modified
Conjugated hydroxycinnamic scaffold	Caffeic acid, chlorogenic acid, rosmarinic acid	Intermediate-to-strong performance with better balance across assays than unsubstituted cinnamic acid	Conjugated side chain and phenolic substitution enhance delocalization of phenoxyl radicals	Promising tunable scaffolds with room for ester/amide or prodrug optimization
Polyfunctional hydroxycinnamic scaffold	Cichoric acid	Excellent ABTS and DPPH activity; solid reducing power	Multiple phenolic sites and extended conjugation support cation-radical scavenging	High-interest lead-relevant scaffold, although complexity and stability require attention
Flavonol scaffold	Quercetin	Among the strongest compounds in ABTS, DPPH, and reducing power	B-ring catechol system and C2=C3 conjugation enhance redox behavior	Strong reference scaffold for medicinal-chemistry translation, but bioavailability remains a limitation
Flavonoid glycoside scaffold	Rutin, eriocitrin	Exceptional ABTS activity with more moderate reducing power	Glycosylation does not abolish radical scavenging, but may alter steric accessibility and transport properties	Interesting for formulation-oriented development rather than direct potency claims

Structural feature	Representative compounds	Typical assay behavior	Chemical interpretation	Physicochemical note
Less hydroxylated flavonoid scaffold	Naringenin, apigenin	Marked assay selectivity; weak DPPH and reducing power	Lower hydroxyl density and altered conjugation reduce radical quenching efficiency	Lower priority unless paired with targeted structural modification

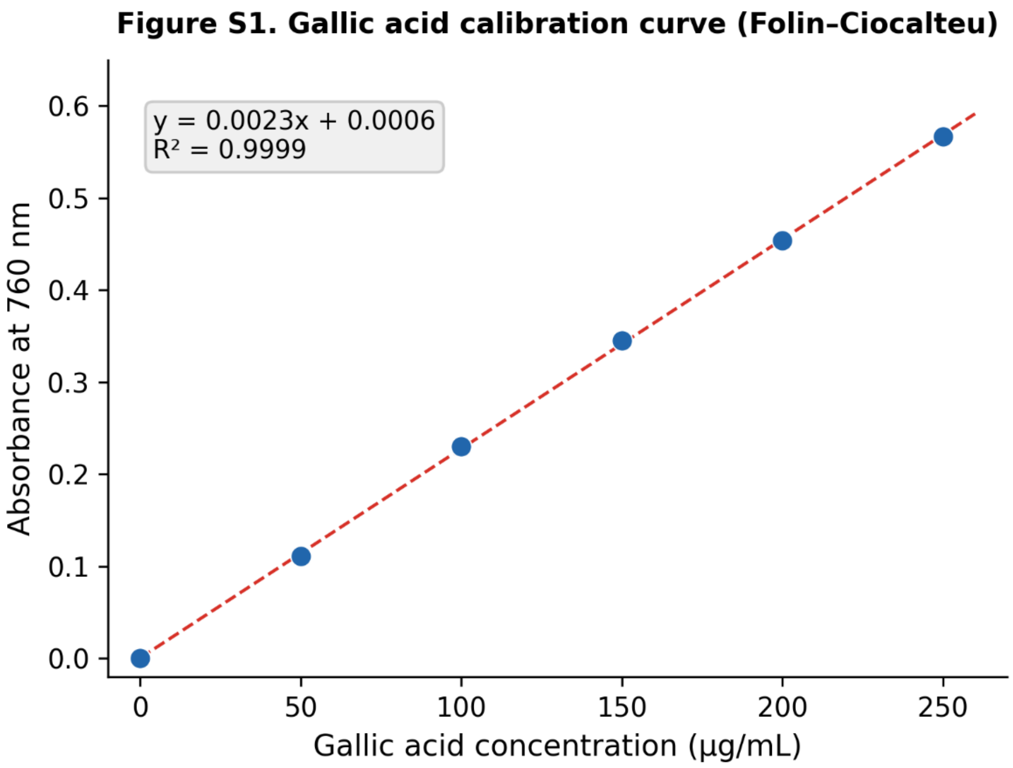

Figure S1. Gallic acid calibration curve for the Folin–Ciocalteu assay. Absorbance was measured at 760 nm against gallic acid concentrations ranging from 0 to 250 µg/mL. The linear regression equation and coefficient of determination (R^2) are shown. All measurements were performed in triplicate.

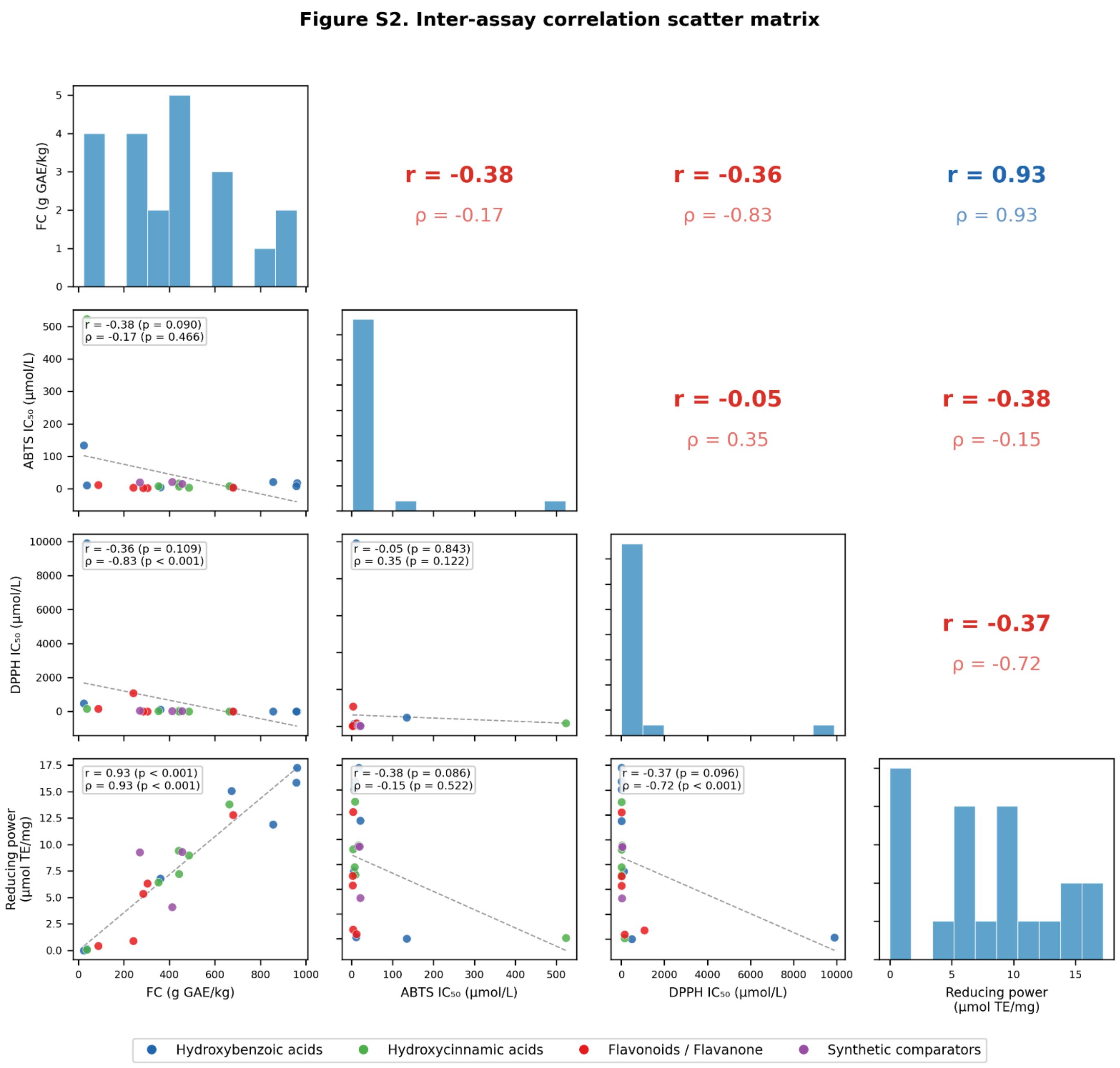

Figure S2. Inter-assay correlation scatter matrix for the four antioxidant endpoints. Lower triangle: scatter plots with Pearson r and Spearman ρ values (with significance levels); diagonal: frequency distributions; upper triangle: correlation coefficients in bold. Data points are color-coded by scaffold class: hydroxybenzoic acids (blue), hydroxycinnamic acids (green), flavonoids/flavanone (red), and synthetic comparators (purple). n = 21 compounds. ***, $p < 0.001$; ns, not significant.

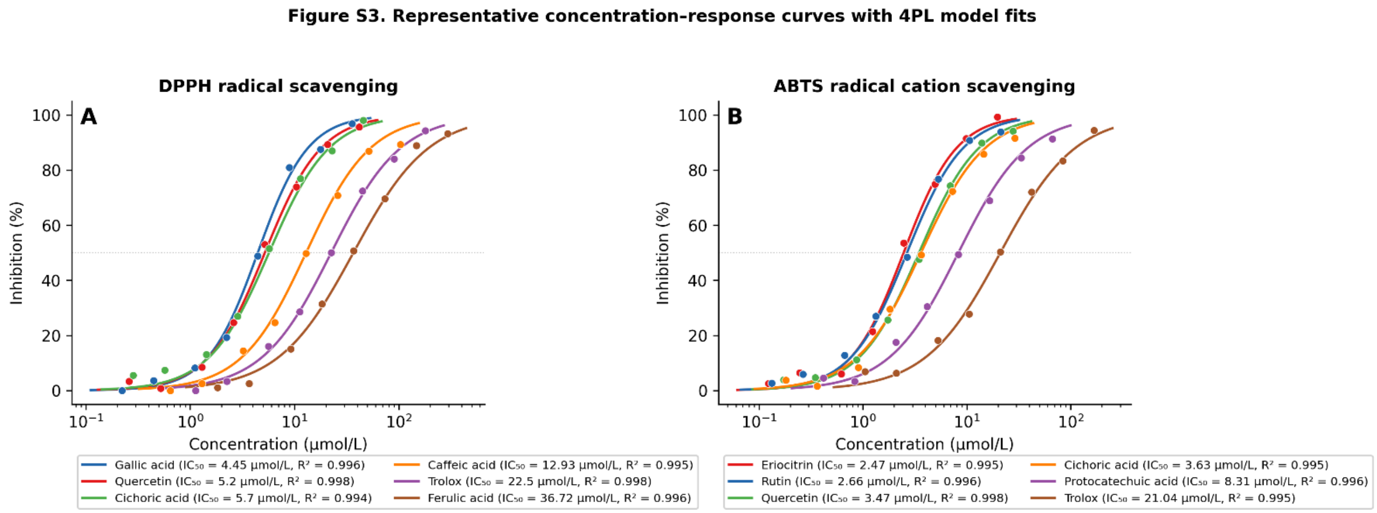

Figure S3. Representative concentration–response curves for selected compounds in (A) DPPH radical scavenging and (B) ABTS radical cation scavenging assays. Curves were fitted using a four-parameter logistic (4PL) sigmoidal model (top constrained to 100%, bottom to 0%, Hill slope unconstrained). IC_{50} values and R^2 for each compound are shown in the legend. The dashed horizontal line indicates 50% inhibition. Compounds were selected to illustrate the range of potencies observed across the dataset.
